# Conserved Odorant-Binding Proteins from Aphids and Eavesdropping Predators

**DOI:** 10.1371/journal.pone.0023608

**Published:** 2011-08-23

**Authors:** Sophie Vandermoten, Frédéric Francis, Eric Haubruge, Walter S. Leal

**Affiliations:** 1 University of Liege, Gembloux Agro-Bio Tech, Functional and Evolutionary Entomology, Gembloux, Belgium; 2 Department of Entomology, University of California Davis, Davis, California, United States of America; Griffith University, Australia

## Abstract

**Background:**

The sesquiterpene (*E*)-ß-farnesene is the main component of the alarm pheromone system of various aphid species studied to date, including the English grain aphid, *Sitobion avenae*. Aphid natural enemies, such as the marmalade hoverfly *Episyrphus balteatus* and the multicolored Asian lady beetle *Harmonia axyridis*, eavesdrop on aphid chemical communication and utilize (*E*)-ß-farnesene as a kairomone to localize their immediate or offspring preys. These aphid-predator systems are important models to study how the olfactory systems of distant insect taxa process the same chemical signal. We postulated that odorant-binding proteins (OBPs), which are highly expressed in insect olfactory tissues and involved in the first step of odorant reception, have conserved regions involved in binding (*E*)-ß-farnesene.

**Methodology:**

We cloned OBP genes from the English grain aphid and two major predators of this aphid species. We then expressed these proteins and compare their binding affinities to the alarm pheromone/kairomone. By using a fluorescence reporter, we tested binding of (*E*)-ß-farnesene and other electrophysiologically and behaviorally active compounds, including a green leaf volatile attractant.

**Conclusion:**

We found that OBPs from disparate taxa of aphids and their predators are highly conserved proteins, with apparently no orthologue genes in other insect species. Properly folded, recombinant proteins from the English grain aphid, SaveOBP3, and the marmalade hoverfly, EbalOBP3, specifically bind (*E*)-ß-farnesene with apparent high affinity. For the first time we have demonstrated that insect species belonging to distinct Orders have conserved OBPs, which specifically bind a common semiochemical and has no binding affinity for related compounds.

## Introduction

Aphids are important agricultural pests throughout the world, with the English grain aphid, *Sitobion avenae* (Hemiptera: Aphididae) being one of the most serious pests of cereals [1]. In the insect's arms race, aphids secrete droplets of a sticky fluid in an attempt to keep parasitoids and predators at bay. The secretion derived from a specialized structure, the cornicles, not only glues mouthparts, antennae, ovipositor and other organs of the attacking enemies, but also contains an alarm pheromone, with (*E*)-ß-farnesene being ubiquitous among Aphidinae species hitherto studied [2]–[5]. This semiochemical may be the sole constituent of the alarm pheromone or it may be the part of a blend that includes monoterpenoid compounds like α-pinene, ß-pinene, and limonene [4], [5]. Alarm pheromone may act as primer or releaser thus eliciting conspecific physiological and behavioral responses, respectively. Thus, they prime an increase proportion of winged morphs in the offspring as well as elicit short-term defensive responses such as feeding cessation and dropping from the host plant [6]–[10]. Parasitoid and predators, on the other hand, eavesdrop on aphid communication and utilize (*E*)-ß-farnesene as a kairomone, which attracts aphid predators and enhance foraging behavior of parasitoids [11]. Electroantennographic recordings from the antennae of a number of aphid predators consistently showed not only significantly higher responses to (*E*)-ß-farnesene than to structural related compounds, but also adaptation to the alarm pheromone [12]–[15]. Additionally, behavioral studies demonstrated that aphid alarm pheromone enhances foraging behavior of hoverflies [14], [16], ground beetle [17], lacewings [15], and lady beetles [13], [18]–[20]. Thus, the aphid-predator systems are important models to study olfaction given that it is hitherto unknown what biochemical machineries insect species in distant taxa use for the reception of a common semiochemical.

Three major olfactory proteins have been demonstrated to be involved in the reception of odorants in insects, namely, odorant-binding proteins (OBPs), odorant receptors (ORs), and odorant-degrading enzymes (ODEs) [21]. OBPs are the liaison between the external environment and ORs. Odorant like pheromones and other semiochemicals reaching the port of entry of olfactory sensilla, the pore tubules, are bound and solubilized by OBPs, transported through the sensillar lymph and the end of the journey released to activate the membrane-bound ORs. After activating ORs stray odorant molecules are inactivated by ODEs [21].

We postulated that OBPs from aphids and their predators might have conserved regions involved in binding (*E*)-ß-farnesene. With the advent of the genome sequence, it has been demonstrated that an OBP from the pea aphid *Acyrthosiphon pisum*, ApisOBP3, specifically bind (*E*)-ß-farnesene [22]. This prompted us to isolate and clone OBPs from another aphid species, the English grain aphid, *Sitobion avenae*, and two predator species in disparate taxa, the multicolored Asian lady beetle, *Harmonia axyridis* (Coleoptera: Coccinellidae) and the marmalade hoverfly, *Episyrphus balteatus* (Diptera: Syrphidae) – the most widely used biological control agent against aphids [23], [24]. Surprisingly, the amino acid sequences of the OBPs from aphids and predators are highly conserved (>90% amino acid identity) with no apparent orthologs in other insect species. Additionally, OBPs from *S. avenae* aphid and *E. balteatus* predator bind the alarm pheromone/kairomone (*E*)-ß-farnesene with apparent high affinity, and discriminate (*E*)-ß-farnesene-related compounds as well as an important plant volatile.

## Results and Discussion

### Cloning of aphid and predator OBPs

**Figure 1:**
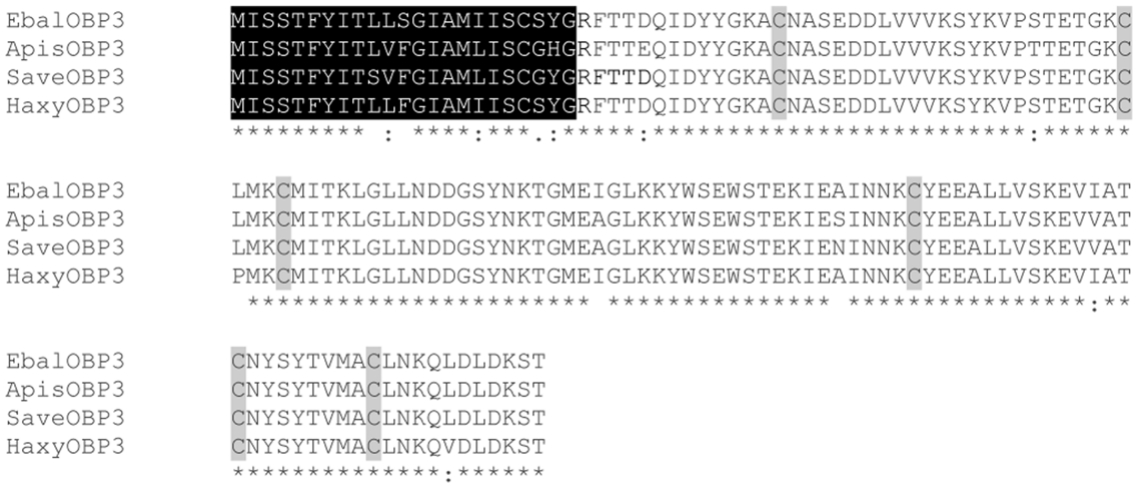
Deduced amino acid sequences from aphids and their predators. CLUSTALX comparison of cDNA coding region of OBP3s from the English grain aphid *S. avenea* (Save), the pea aphid *A. piusm* (Apis), the marmalade hoverfly *E. balteatus* (Ebal), and the multicolored Asian lady beetle, *H. axyridis* (Haxy). The sequences of the predicted signal peptides are highlighted in black boxes. The conserved cysteine residues are denoted by grey boxes. Identities between each pair of OBP3s are between 94 and 98%.

Our cloning approach led to the amplification of four separate cDNAs, one from the English grain aphid, *S. avenae*, two from the marmalade hoverfly *E. balteatus*, and one from the multicolored Asian lady beetle, *H. axyridis*, but the two *E. balteatus* isoforms from male and female antennae encoded the same protein. Because the encoded proteins share high amino acid identity to the pea aphid ApisOBP3 (Fig. 1), we named the newly identified OBPs SaveOBP3, EbalOBP3, and HaxyOBP3, respectively.

As expected for OBPs, the N-terminal sequences (23 amino acid residues) were predicted by SIGNALP server to be signal peptides. The theoretical MW deduced from putative amino acid sequences of mature SaveOBP3, EbalOBP3, and HaxyOBP3 were 15.78, 15.84, and 15.81 kDa respectively, which is a typical MW size for insect OBPs [25]. Like the vast majority of insect OBPs, the newly identified OBPs are acidic proteins, with a calculated isoelectric points (pI) of 5.17. In another hallmark of insect OBPs, SaveOBP3, EbalOPB3, HaxyOBP3, share six well-conserved cysteine residues - a feature of “Classic” OBPs [26] (Fig. 1). Interestingly, no apparent orthologs have been found by BLASPp analysis. Moreover, a CLUSTALX alignment revealed that the amino acid sequences show more than 90% similarity to *A. pisum* ApisOBP3 [22], with proteins from such distant taxa differing only in 2–6 amino acid residues.

### Functional expression

**Figure 2:**
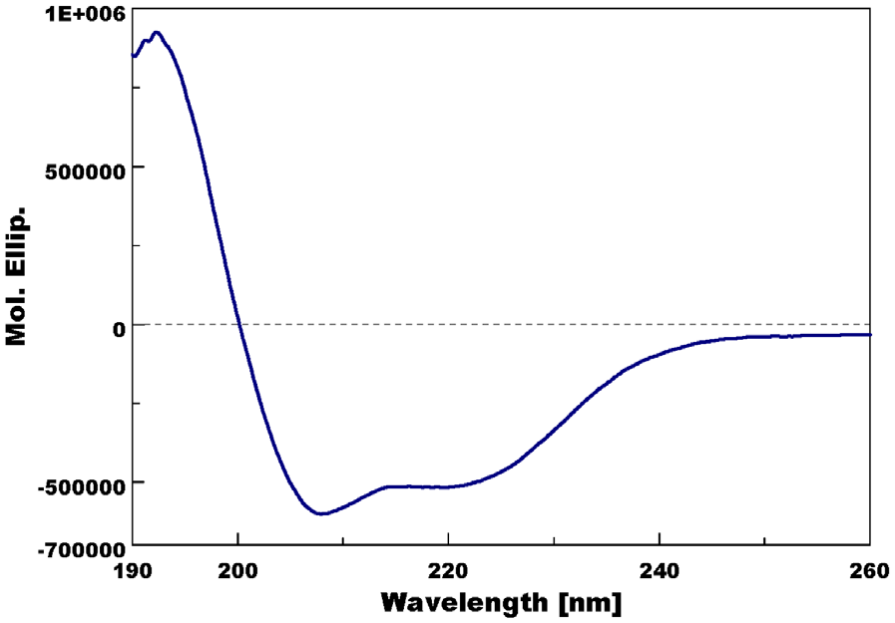
Far-UV CD spectrum of EbalOPB3. The two minima and a maximum of the spectrum at pH 7 suggest that EbalOBP3 is a properly folded α-helical-rich OBP.

Using a perisplamic expression system known to generate properly folded, functional OBPs [27], we generated samples of recombinant EbalOBP3, SaveOBP3, and HaxyOBP3. The cDNAs encoding the three mature OBPs were subcloned in pET-22b(+), and BL21 (DE3) cells were transformed with the recombinant vectors for large scale expressions. Purification by a combination of ion-exchange chromatography and gel filtration generated pure samples (>99%) of EbalOBP3 and SaveOBP3. Because of low purity, samples of HaxyOBP3 were not used for further studies. Circular dichroism (CD) analysis suggested that pure proteins were properly folded. As shown for EbalOPB3 (Fig. 2), the far-UV CD spectrum showed a maximum at 193 nm and two minima at 208 and 220 nm, a typical profile of α-helical-rich OBPs [28]–[34].

### Binding assays

**Figure 3:**
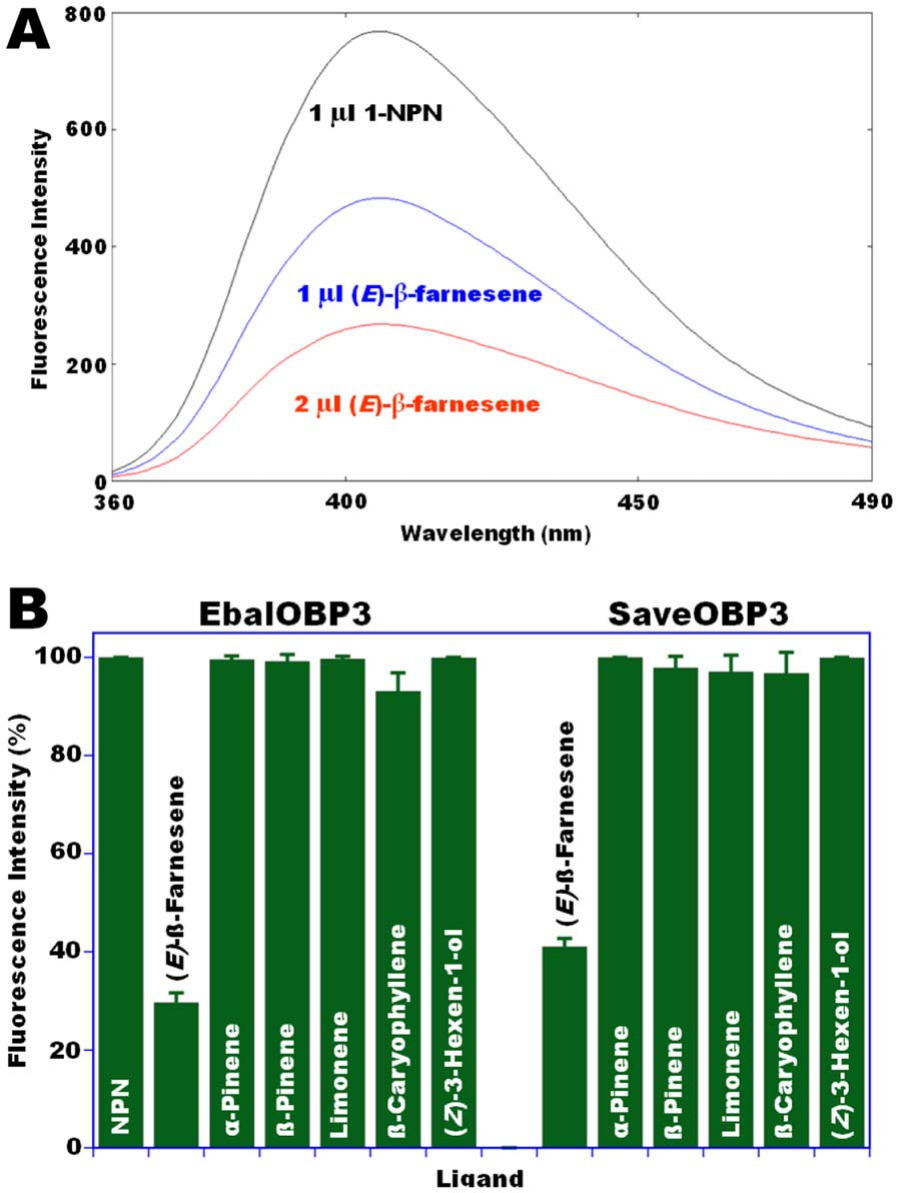
Competitive binding of (*E*)-β-farnesene to OBP3s. (**A**) Fluorescence emission spectra of SaveOBP3 (10 µg/ml, pH 7) in the presence of NPN (1 µl, 1.6 µM; black line), and after titrating with increasing amounts of (*E*)-β-farnesene (1 µl or 1.6 µM: blue line; 2 µl or 3.2 µM: red line). Replacement of the fluorescent reporter is indicated by quenching (decrease in fluorescence emission) thus suggesting a higher affinity for the semiochemical. (**B**) When challenged with various semiochemicals both EbalOBP3 and SaveOBP3 showed specific affinity for (*E*)-β-farnesene. Error bars show standard deviation.

Having observed that EbalOBP3 and SaveOBP3 were properly folded, we next assessed by a competitive assay using NPN as a fluorescence reporter [35], [36] the affinity of these OBPs for (*E*)-ß-farnesene, which is an alarm pheromone for the English grain aphid and a kairomone for its predator, the marmalade hoverfly. We tested also other ecologically significant compounds, namely, α-pinene, β-pinene, limonene, β-caryophyllene, and (Z)-3-hexen-1-ol. These compounds have been demonstrated to be electrophysiologically and behaviorally active. In addition, α-pinene, β-pinene, and limonene are secondary constituents of the alarm pheromone system of various aphid species, and the green leaf volatile (Z)-3-hexen-1-ol increased mobility of females of the marmalade hoverfly, plant acceptance, and oviposition activity even in the absence of prey [14]. Both SaveOBP3 (Fig. 3A,B) and EbalOBP3 (Fig. 3B) bound (*E*)-ß-farnesene with apparent high affinity. By contrast none of the other tested compound bound to SaveOBP3 or EbalOBP3 (Fig. 3B). This is, therefore, the first demonstration that OBPs from an aphid and its predator from a distant taxa (Homoptera vs. Diptera) specifically bind a semiochemical, which aphids utilize for intraspecific communication as an alarm pheromone and the hoverflies eavesdrop to find sites for laying eggs.

### Conclusion

Whole genome surveys have shown that OBPs are highly divergent protein families and are characterized by lineage-specific expansions, presumably driven largely by adaptation [35], [37]. Thus, it is highly surprising that the three OBPs reported here and identified from species belonging to three distinct insect Orders, i.e. Homoptera, Coleoptera, and Diptera have remarkably high percentage identity: SaveOBP3 vs EbalOBP3, 94%; similarity, 96%, SaveOBP3 vs HaxyOBP3, 94%; similarity, 96%, and EbalOBP3 vs HaxyOBP3, 98%; similarity 99%. These observations, in addition to the absence of orthologous genes among other insect species, strongly support the hypothesis that the newly identified OBP3 are indeed involved in semiochemical reception. Our fluorescence-based binding assays revealed that both SaveOBP3 and EbalOBP3 are specifically tuned to (*E*)-β-farnesene. To the best of our knowledge, this is the first evidence that preys and predators utilize highly conserved olfactory proteins for the recognition of a common and ecologically significant chemical signal. Interestingly, both male and female hoverflies express EbalOBP3. While detection of an aphid-derived semiochemical by female hoverflies is essential for offspring survival, males may hone in for mate finding as it is known that males wait near potential oviposition sites for potential mates [38]. Location of suitable sites for oviposition by gravid female hoverflies is essential for offspring survival as larvae are aphid predators.

Our findings have also practical applications as the OBPs reported here may serve as molecular target for the development of eco-friendly strategies for management of aphid populations. These molecular targets may lead to compounds that augment biological control by facilitating host finding by the predators and/or by disrupting aphid chemical communication.

## Materials and Methods

### Insect rearing

Pupae of *E. balteatus* were purchased from Katz Biotech AG (Baruth, Germany). Hoverflies were reared with sugar, pollen and water in a climate-controlled room (16 h light photoperiod; 60±5% RH; 20±2°C). Male and female flies were separated for further dissection. The English grain aphid *S. avenae* was reared on wheat plants in a dedicated environmental chamber operated at 20±2°C, under a 16 h light photoperiod. A lab colony of *H. axyridis* was derived from individuals collected in Gembloux, Belgium during fall 2009. The larvae and the resulting adults were provisioned daily *ad libitum* with aphids, *Acyrthosiphon pisum*, which was reared on beans *Vicia faba*. Sugar, multiflower pollen and water were also provided. Boxes were placed in controlled environment incubators (16 h light photoperiod; 25±2°C; 60±5% RH).

### cDNA synthesis and *OBP* cloning

Total RNA was isolated from antennae using the RNeasy Mini Kit (Qiagen, Venlo, Netherlands) following the manufacturer's instructions. Dissections were performed on 80–100 individuals of hoverflies and ladybirds and 150–200 individuals of aphids. First-strand cDNA was synthesized from 1 µg total RNA using an oligo-dT primer per manufacturer's instructions provided with the RevertAid™ First Strand cDNA Synthesis Kit (Fermentas, St. Leon-Rot, Germany). A Homology cloning strategy was used to identify OBP3 genes. First, to amplify a core fragment, a set of specific and degenerate primers were designed according to the known cDNA sequence of *A. pisum* OBP3 (ApisOBP3) (Pspec1-Fwd 5′- GAT TAT TAT GGA AAA GCG TGC AAC GCC AGC -3′; Pspec2-Rev: 5′- AAC GAC GAT GGT TCG TAC AAC AAA ACT GGC ATG -3′; Pdeg2-Rev: 5′- TCG YAY RMN NKN AWR GCA TGT -3′; Pdeg1-Rev: RTN GCG ACG TGC ARY WAN TCG YAY -3′). Given the high level of nucleic acid identity between the sequences obtained, primers specific to the N-terminal (P1-Fwd: 5′- ATG ATT TCG TCG ACG TTT TAC ATA ACG -3′) and C-terminal (P2-Rev: 5′- TTG GAT CTC GAC AAG TCA ACT TGA) sequences of ApisOBP3 were designed. One fourth of the reverse transcription reaction was used for PCR amplification with 1 µM of reverse and forward primers, 0.2 mM of each dNTP in 1× Dream Taq PCR buffer (Fermentas, St. Leon-Rot, Germany) and 1.5 units of Dream Taq (Fermentas, St. Leon-Rot, Germany). The PCR cycling conditions was carried out as follows: a first denaturation step at 95°C for 3 min followed by 35 cycles of 95°C, 45 s; 55°C, 45 s; 72°C, 1 min; and a final extension step at 72°C for 5 min. Using the QIAEXII Gel Extraction Kit (Qiagen, Venlo, Netherlands), the bands were purified from the agarose gel, cloned in the pTZ57R/T vector (InsTAclone™ PCR Cloning Kit, Fermentas, St. Leon-Rot, Germany) according to the manufacturer's instructions, and sequenced. The putative signal peptides and most likely cleavage sites were predicted by using the SIGNALP 3.0 server [39]. We included all the sequences reported here in GenBank (HQ896240, HaxyOBP3; HQ89624, EbalOBP3; and HQ896243, SaveOBP3). Sequence data were aligned using CLUSTALX [40].

### Recombinant protein expression and purification

One microgram of pET-22b(+) vector (EMD Chemicals, Gibbstown, NJ) was digested with 2.5 U of Msc I (Fermentas, St. Leon-Rot, Germany) at 37°C for 90 min. After purification of DNA by GeneJet PCR Purification kit (Fermentas, St. Leon-Rot, Germany) the vector was digested with 5 U of Bam HI (Fermentas, St. Leon-Rot, Germany) at 37°C for 90 min and subsequently gel-purified by QIAEXII Gel Extraction Kit (Qiagen, Venlo, Netherlands). The following primers were used for amplification of insert DNA in which the signal peptide was removed: OBPpET22-FWD2 (TTA TAG AGC TCC CGA TTT ACG AC**A** GAT C) for cloning of EbalOBP3, HaxyOBP3; OBPpET22_FWD3 (TTA TAG AGC TCC CGA TTT ACG ACG GAT C -3′) for cloning of SaveOBP3; and the reverse primer (5′- CGC GGA TCC TCA AGT TGA CTT GTC GAG ATC -3′). Cutting sites for Sac I (forward primers) and BamH I (reverse primer) restriction enzymes are underlined. The PCR product was first cloned into the pTZ57R/T vector (InsTAclone™ PCR Cloning Kit, Fermentas, St. Leon-Rot, Germany). After amplification, and confirmation by sequencing, 2 µg of plasmid were initially digested with 10 U of Sac I (Fermentas, St. Leon-Rot, Germany) at 37°C for 150 min, purified by GeneJet PCR Purification kit (Fermentas, St. Leon-Rot, Germany), blunted by T4 DNA polymerase (Fermentas, St. Leon-Rot, Germany) with dNTP, and purified again by GeneJet PCR Purification kit (Fermentas, St. Leon-Rot, Germany). Then, the DNA was digested with 10 U of BamH I at 37°C for 90 min and, gel-purified by QIAEXII Gel Extraction Kit (Qiagen, Venlo, Netherlands), and ligated into prepared pET-22b(+) vector following the manufacturer's instructions provided with the Rapid DNA Ligation kit (Roche Applied Science, Vilvoorde, Belgium).

### Protein expression and purification

Expression was performed in LB medium with transformed BL21 (DE3) cells (Novagen, San Diego, CA). Proteins in the periplasmic fraction were extracted with 10 mM Tris-HCl (pH 8) by using four cycles of freeze-and-thaw and centrifuging at 16,000× g to remove debris. The supernatant was loaded on a HiprepTM DEAE 16/10 column (GE Healthcare, Piscataway, NJ) and separated with a linear gradient of 0–500 mM NaCl in 10 mM Tris-HCl (pH 8). Fractions were analyzed by SDS-PAGE and those containing the target protein were further purified on a Superdex-75 26/60 gel-filtration column (GE Healthcare, Piscataway, NJ) pre-equilibrated with 150 mM NaCl and 20 mM Tris-HCl (pH 8). Highly purified protein fractions were desalted on HiTrap desalting column by using water as mobile phase. The concentrations of the recombinant proteins were measured by UV at 280 nm in 20 mM sodium phosphate (pH 6.5) and 6 M guanidine HCl by using the theoretical extinction coefficient calculated with EXPASY software (http://us.expasy.org/tools/protparam.html).

### Fluorescence binding assay

N-phenyl-1-naphthylamine (NPN) was used as a reporter ligand in fluorescence binding assay experiments [35]. First, the affinities of NPN to recombinant OBP3s were measured using 10 µg/ml protein solutions prepared in 20 mM ammonium acetate, pH 7. For all the protein tested, 1.6 µM final concentration of NPN was added to reach fluorescence intensity saturation, which was used as a reference (100%) to normalize the following measurements. Then, one of the selected ligands was added (1.6 µM final concentrations) and the fluorescence intensities were recorded and normalized by using the NPN reference. Fluorescence measurements were done on a spectrofluorophotometer (RF-5301, Shimadzu, Kyoto, Japan). Samples in 2-ml cell were excited at 337 nm, and the emission spectra were recorded from 340 to 500 nm, with emission and excitation slit widths of 1.5 and 10 nm, respectively.

### Circular dichroism (CD) detection

CD experiments were performed on J-810 spectropolarimeter (Jasco, Easton, MD). The CD spectra were recorded from 185 to 260 nm of wavelength with 1 nm resolution and 4 s of average time. A small amount of the recombinant OBP3 (final concentration, 0.2 mg/ml) was diluted in 20 mM ammonium acetate, pH 7.

## References

[1] Blackman RL, Eastop VF (2006). Aphids on the world herbaceous plants and shrubs: An identification and information guide.

[2] Bowers WS, Nault LR, Webb RE, Dutky SR (1972). Aphid alarm pheromone: Isolation, identification, synthesis.. Science.

[3] Wientjens WH, Lakwijk AC, Vanderma T (1973). Alarm pheromone of grain aphids.. Experientia.

[4] Pickett JA, Griffith DC (1980). Composition of aphid alarm pheromones.. J Chem Ecol.

[5] Francis F, Vandermoten S, Verheggen F, Lognay G, Haubruge E (2005). Is the (E)-ß-farnesene only volatile terpenoid in aphids?. J Appl Entomol.

[6] Hatano E, Kunert G, Weisser WW (2010). Aphid wing induction and ecological costs of alarm pheromone emission under field conditions.. Plos One.

[7] Kislow C, Edwards LJ (1972). Repellent odour in aphids.. Nature.

[8] Kunert G, Otto S, Röse US, Gershenzon J, Weisser WW (2005). Alarm pheromone mediates production of winged dispersal morphs in aphids.. Ecol Lett.

[9] Pickett JA, Wadhams LJ, Woodcock CM (1992). The chemical ecology of aphids.. Annu Rev Entomol.

[10] Podjasek JO, Bosnjak LM, Brooker DJ, Mondor EB (2005). Alarm pheromone induces a transgenerational wing polyphenism in the pea aphid, *Acyrthosiphon pisum*.. Can J Zool.

[11] Hatano E, Kunert G, Michaud JP, Weisser WW (2008). Chemical cues mediating aphid location by natural enemies.. Eur J Entomol.

[12] Al Abassi S, Birkett MA, Pettersson J, Pickett JA, Wadhams LJ (2000). Response of the seven-spot ladybird to an aphid alarm pheromone and an alarm pheromone inhibitor is mediated by paired olfactory cells.. J Chem Ecol.

[13] Verheggen FJ, Fagel Q, Heuskin S, Lognay G, Francis F (2007). Electrophysiological and behavioral responses of the multicolored asian lady beetle, *Harmonia axyridis* (Pallas), to sesquiterpene semiochemicals.. J Chem Ecol.

[14] Verheggen FJ, Arnaud L, Bartram S, Gohy M, Haubruge E (2008). Aphid and plant volatiles induce oviposition in an aphidophagous hoverfly.. J Chem Ecol.

[15] Zhu JW, Cossé AA, Obrycki JJ, Boo KS, Baker TC (1999). Olfactory reactions of the twelve-spotted lady beetle, *Coleomegilla maculata* and the green lacewing, *Chrysoperla carnea* to semiochemicals released from their prey and host plant: electroantennogram and behavioral responses.. J Chem Ecol.

[16] Francis F, Martin T, Lognay G, Haubruge E (2005). Role of (E)-ß-farnesene in systematic aphid prey location by *Episyrphus balteatus* larvae (Diptera: Syrphidae).. Eur J Entomol.

[17] Kielty JP, Allen-Williams LJ, Underwood N, Eastwood EA (1996). Behavioral responses of three species of ground beetle (Coleoptera: Carabidae) to olfactory cues associated with prey and habitat.. J Insect Behav.

[18] Acar EB, Medina JC, Lee ML, Booth GM (2001). Olfactory behavior of convergent lady beetles (Coleoptera: Coccinellidae) to alarm pheromone of green peach aphid (Hemiptera: Aphididae).. Can Entomologist.

[19] Francis F, Lognay G, Haubruge E (2004). Olfactory responses to aphid and host plant volatile releases: (E)-β-Farnesene an effective kairomone for the predator *Adalia bipunctata*.. J Chem Ecol.

[20] Nakamuta K (1991). Aphid alarm pheromone component, (E)-beta-farnesene, and local search by a predatory lady beetle, *Coccinella septempunctata* Bruckii mulsant (Coleoptera, Coccinellidae).. Appl Entomol Zool.

[21] Vogt RG, Gilbert LI, Iatro K, Gill S (2005). Molecular basis of pheromone detection in insects.. Comprehensive Insect Physiology, Biochemistry, Pharmacology and Molecular Biology.

[22] Qiao H, Tuccori E, He X, Gazzano A, Field L (2009). Discrimination of alarm pheromone (E)-beta-farnesene by aphid odorant-binding proteins.. Insect Biochem Mol Biol.

[23] Chambers RJ, Adams THL (1986). Quantification of the impact of hoverflies (Diptera: Syrphidae) on cereal aphids in winter wheat: An analysis of field populations.. J Appl Ecol.

[24] Tenhumberg B, Poehling H-M (1995). Syrphids as natural enemies of cereal aphids in Germany: aspects of their biology and efficacy in different years and regions.. Agric Ecosyst Environ.

[25] Yang G, Winberg G, Ren H, Zhang S (2011). Expression, purification and functional analysis of an odorant binding protein AaegOBP22 from *Aedes aegypti*.. Protein Exp Purif.

[26] Leal WS, Blomquist GJ, Vogt RG (2003). Proteins that make sense.. Insect pheromone biochemistry and molecular biology, the biosynthesis and detection of pheromone and plant volatiles.

[27] Wojtasek H, Leal WS (1999). Conformational change in the pheromone-binding protein from *Bombyx mori* induced by pH and by interaction with membranes.. J Biol Chem.

[28] Damberger FF, Ishida Y, Leal WS, Wüthrich K (2007). Structural basis of ligand binding and release in insect pheromone-binding proteins: NMR structure of antheraea polyphemus PBP1 at pH 4.5.. J Mol Biol.

[29] Horst R, Damberger F, Luginbühl P, Güntert P, Peng G (2001). NMR structure reveals intramolecular regulation mechanism for pheromone binding and release.. Proc Natl Acad Sci, USA.

[30] Lartigue A, Gruez A, Briand L, Blon F, Bézirard V (2004). Sulfur single-wavelength anomalous diffraction crystal structure of a pheromone-binding protein from the honeybee *Apis mellifera* L.. J Biol Chem.

[31] Lartigue A, Gruez A, Spinelli S, Rivière S, Brossut R (2003). The crystal structure of a cockroach pheromone-binding protein suggests a new ligand binding and release mechanism.. J Biol Chem.

[32] Leal WS, Nikonova L, Peng G (1999). Disulfide structure of the pheromone binding protein from the silkworm moth, *Bombyx mori*.. FEBS Letters.

[33] Scaloni A, Monti M, Angeli S, Pelosi P (1999). Structural analysis and disulfide-bridge pairing of two odorant-binding proteins from *Bombyx mori*.. Biochem Biophys Res Commun.

[34] Xu W, Cornel AJ, Leal WS (2010). Odorant-binding proteins of the malaria mosquito *Anopheles funestus* sensu stricto.. Plos One.

[35] Pelosi P, Zhou JJ, Ban LP, Calvello M (2006). Soluble proteins in insect chemical communication.. Cell Mol Life Sci.

[36] Campanacci V, Krieger J, Bette S, Sturgis JN, Lartigue A (2001). Revisiting the specificity of *Mamestra brassicae* and *Antheraea polyphemus* pheromone-binding proteins with a fluorescence binding assay.. J Biol Chem.

[37] Zhou J-J, Field LM, He XL (2009). Insect Odorant-Binding Proteins: Do They Offer an Alternative Pest Control Strategy?. Outlooks on Pest Management.

[38] Maier CT, Waldbauer GP (1979). Dual mate-seeking strategies in male syrphid flies (Diptera: Syrphidae).. Ann Entomol Soc Am.

[39] Bendtsen JD, Nielsen H, von Heijne G, Brunak S (2004). Improved prediction of signal peptides: SignalP 3.0.. J Mol Biol.

[40] Thompson JD, Higgins DJ, Gibson TJ (1994). CLUSTAL W: improving the sensitivity of progressive multiple sequence alignment through sequence weighting, position-specific gap penalties and weight matrix choice.. Nucleic Acids Res.

