# Correction: Conserved Odorant-Binding Proteins from Aphids and Eavesdropping Predators

**DOI:** 10.1371/annotation/6fb8a803-8203-429c-b0d1-4d0d995c39e9

**Published:** 2012-12-11

**Authors:** Sophie Vandermoten, Frédéric Francis, Eric Haubruge, Walter S. Leal

The authors have become aware of concerns over the reliability of some of the results reported in the article.

Figure 1 shows orthologs of SaveOBP3 and ApisOBP3 in the multicolored Asian lady bug Harmonia axyridis (HaxyOBP3) and in the marmalade hoverfly Episyrphus balteatus (EbalOBP3). The cDNAs encoding these proteins were cloned in the Belgium laboratory at the University of Liege and subsequent parts of the work, specifically protein expression, biochemical and biophysical studies and binding assays, were performed in the UC Davis laboratory. Recently, we attempted to re-clone HaxyOBP3 at UC Davis and were surprised that our attempts were unrewarding. We were also unable to clone the HaxyOBP3 cDNA using samples from different locations. Additionally, we performed Western blots using an aphid OBP3 antibody (received from Paolo Pelosi, University of Pisa, Italy) and found no evidence of HaxyOBP3 being expressed in the beetle antennae. We have also tried similar attempts with EbalOPB3 and were unable to clone EbalOBP3 cDNA or find the putative protein band.

We therefore conclude that the "orthologs" from beetle (HaxyOBP3) and the hoverfly (EbalOBP3) reported in the article are artifacts, probably derived from the template from the European grain aphid, Sitobion avenae.

In conclusion, Figure 1 in the article is inaccurate and its results should be disregarded. The expression of the EbalOBP3 recombinant protein and the data presented in Figures 2 and 3 are accurate, however, readers should be aware that the EbalOBP3 gene reported is likely chimeric, and thus that the results do not represent proteins naturally expressed in the antennae of the marmalade hoverfly. 

